# S133. THE RELATIONSHIP BETWEEN EXPOSURE TO TRAUMA DURING CHILDHOOD AND ADOLESCENCE AND PSYCHOTIC EXPERIENCES AT 18 YEARS OLD IN A NON-CLINICAL POPULATION

**DOI:** 10.1093/schbul/sby018.920

**Published:** 2018-04-01

**Authors:** Jazz Croft, Stanley Zammit, Jon Heron, Christoph Teufel

**Affiliations:** 1 University of Bristol; 2 University of Bristol, Cardiff University; 3 Cardiff University

## Abstract

**Background:**

Childhood trauma (CT) is an established risk factor for the onset and development of psychotic experiences (PEs), with some evidence that interpersonal traumas being more strongly associated with risk of PEs than accidental injury or adversities such as parental divorce. However, it is not clear whether specific types of interpersonal trauma, such as emotional neglect or sexual abuse, are more strongly associated with risk of PEs than others, or whether there are critical or sensitive periods within which exposure to these carries the greatest risk.

**Methods:**

Our study aimed to extend this literature by comparing the presence of multiple exposures to identify the most important types of trauma exposure and examining the association of trauma exposure with PEs throughout childhood in a large sample. We investigated whether different trauma exposures at different points of development from 0–17 years old are associated with PEs in The Avon Longitudinal Study of Parents and Children (ALSPAC) birth cohort. The measures of different types of trauma (physical abuse, emotional abuse, bullying, domestic violence, sexual abuse, emotional neglect) at different time-points (0–5 years old, 5–11 years old, 11–17 years old) were derived from questions asked throughout the longitudinal study. PEs were assessed by the face-to-face, semi-structured, Psychosis-Like Symptoms interview (PLIKSi) at 18 years old. The primary outcome was suspected or definite psychotic experiences (PEs). Multivariable logistic regression was used to examine the effects of exposure to specific types of trauma, and of exposure to trauma across specific age periods, before and after adjusting for the presence of other traumatic exposures and for confounders, including measures of socio-economic measures (parental income, maternal education, crowded living conditions at birth), sex, measures of parental mental health, parental drug use, parental trouble with the law, IQ and genetic risk for schizophrenia, bipolar disorder and major depressive disorder. The robustness of these findings will be explored by using penalised-regression and MI methods to test specific competing hypotheses and address potential bias due to missing data.

**Results:**

All trauma types exposed to between 0–17 years of age were strongly associated with PEs (adjusted ORs 1.64 – 2.13). When controlling for the presence of other trauma exposures and confounders, exposure to domestic violence at 0–5 (adjusted OR 1.67, 95% CI 1.24 – 2.26), bullying at 5–11 (adjusted OR 1.83, 95% CI 1.34, 2.48) and sexual abuse, emotional neglect and bullying at 11–17 (adjusted ORs 2.04 – 2.39) were the traumas most strongly associated with PEs (p<.001). Whilst exposure to any trauma at each of the age-periods was associated with PEs (adjusted ORs 1.55–2.11), the strongest association was for exposure at ages 11–17 years. The cumulative risk of reporting multiple types trauma exposures at each time-point further increased the risk of reporting PEs. Adjusting for confounders did not alter the association between trauma exposures and PEs.

**Discussion:**

Previous studies have shown that different types of trauma during childhood increase the risk of PEs. Our results show that the association between trauma and PEs is not explained by a broad range of potential confounders, including genetic risk for schizophrenia, supporting a causal effect of trauma exposure on psychosis. Our findings do not support the presence of a critical period of risk, but indicate that exposure to trauma during any period of childhood or adolescent development increases risk of psychotic experiences.

